# QTL mapping and identification of genes associated with the resistance to *Acanthoscelides obtectus* in cultivated common bean using a high-density genetic linkage map

**DOI:** 10.1186/s12870-022-03635-4

**Published:** 2022-05-25

**Authors:** Xiaoming Li, Yongsheng Tang, Lanfen Wang, Yujie Chang, Jing Wu, Shumin Wang

**Affiliations:** 1 Institute of Crop Science, Chinese Academy of Agricultural Sciences, Beijing, 100081 China; 2 Qujing Academy of Agricultural Sciences, Qujing, 655000 China

**Keywords:** Common bean, *Acanthoscelides obtectus*, Bruchid resistance, High-density genetic linkage map, QTL

## Abstract

**Background:**

Common bean (*Phaseolus vulgaris* L.) is an important agricultural product with large nutritional value, and the insect pest *Acanthoscelides obtectus* (Say) seriously affects its product quality and commodity quality during storage. Few researches on genes of bruchid resistance have investigated in common bean cultivars.

**Results:**

In this study, a bruchid-resistant cultivar black kidney bean and a highly susceptible accession Longyundou3 from different gene banks were crossed to construct a recombinant inbred line population. The genetic analysis indicated a quantitative inheritance of the bruchid resistance trait controlled by polygenes. A high-density genetic map of a total map distance of 1283.68 cM with an average interval of 0.61 cM between each marker was constructed using an F_6_ population of 157 recombinant inbred lines. The map has 3106 bin markers, containing 2,234,769 SNPs. Using the high-density genetic map, a new quantitative trait locus for the resistance to *Acanthoscelides obtectus* was identified on chromosome 6. New molecular markers based on the candidate region were developed, and this locus was further delimited to an interval of 122.3 kb between SSR markers I6–4 and I6–16 using an F_2_ population. This region comprised five genes. *Phvul.006G003700*, which encodes a bifunctional inhibitor, may be a potential candidate gene for bruchid resistance. Sequencing analysis of candidate gene identified a 5 bp insertion-deletion in promoter of gene *Phvul.006G003700* between two parents. Expression analysis of candidate gene revealed that the expression level of *Phvul.006G003700* in bruchid-resistant parent was markedly higher than that in bruchid-susceptible parent both in dry seeds and leaves.

**Conclusions:**

A high-density genetic linkage map was constructed utilizing whole-genome resequencing and one new QTL for bruchid resistance was identified on chromosome 6 in common bean cultivar. *Phvul.006G003700* (encoding a bifunctional inhibitor) may be a potential candidate gene. These results may form the basis for further research to reveal the bruchid resistance molecular mechanism of common bean.

**Supplementary Information:**

The online version contains supplementary material available at 10.1186/s12870-022-03635-4.

## Background

Common bean (*Phaseolus vulgaris* L.) is one of the most widely adapted legumes in the developing world and the most important food legume for direct human consumption in the world [1, 2]. This autodiploid (2n = 2x = 22) species has 11 chromosomes and a genome size of about 537 Mb (*Phaseolus vulgaris* v2.1, DOE-JGI and USDA-NIFA, http://phytozome.jgi.doe.gov/). Common bean is an annual and self-pollinating plant, and its seeds are rich in protein, amino acids, flavonoids, alkaloids and terpenoids, which are necessary for human life [3–6]. Common bean originated from Mesoamerica, and China is considered as a secondary centre of common bean diversity and has a long history of cultivation [7, 8]. Common bean is an important source of plant protein in the human dietary structure and is one of the main export products of China [9].

During their storage, legume seeds are often damaged by legume weevils, which lead to a serious decrease in yield. Bean weevils [*Acanthoscelides obtectus* (Say)] and Mexican bean weevils [*Zabrotes subfasciatus* (Boheman)] are the main insect pests of common bean during storage [10, 11]. *Acanthoscelides obtectus* (Say) originated from South America and Central America and has been introduced into dozens of countries and regions, such as Afghanistan, Japan, Myanmar and China, through international trade and introduction channels [12]. The larvae of *A. obtectus* can enter the bean seeds for feeding. The adults fly to the fields or warehouses and continue to damage common bean, causing great losses [13, 14]. The damage and weight loss rates of common bean in the field are 30% ~ 38% and 6% ~ 8%, respectively, and storage increases these values to 74% and 64%, respectively, which indicates that the damage created by bruchids is more serious during the storage period [15–17]. At present, the commonly used physical methods, including freezing [18–20], boiling and seed dressing treatment [21] as well as chemical methods of fumigation with chemical agents such as phosphine [22] and ethyl formate [23], and biological control [24, 25] are applied to control seeds damage by bruchids, but the negative impacts on the seeds themselves and the environment are substantial.

Because the growth and development of insects are affected by the chemical components in plants [26], which can kill, resist, avoid insects or inhibit their growth, the direct utilization of plants themselves to prevent and control pests has become a research hotspot. Many common bean varieties are resistant to azuki bean weevils (*Callosobruchus chinensis*) and cowpea weevils (*Callosobruchus maculatus*) because they contain special seed proteins [27]. Theses proteins are components of a polygene family encoded by APA locus [arcelin (Arc), phytohemagglutinin (PHA), and α-amylase inhibitor (αAI)] and are most abundant in PHA and αAI [28–33]. PHA and αAI are the most representative members of the lectin family and normally occur in wild and cultivated accessions of common bean. Arcelin is found only in some wild Mexican bean varieties but not in cultivated bean varieties [34–36]. The wild common bean germplasms G02771 (containing ARC-5 protein) [37], G12882 (including ARC-1) [33], RAZ106 (containing ARC-1), G12866 (including ARC-2) [38], G24582 (including ARC-7), G24582A (including ARC-7) and G24584 (including ARC-7) [39] are effective against the Mexican bean weevils. The wild bean germplasms G12952 (ARC-4), G12949 (ARC-4) [32] and QUES (ARC-8) [40] are resistant to both *A. obtectus* and *Z. subfasciatus*. Baldin et al. [41] screened 18 common bean genotypes of Andean American and Mesoamerican origin, and the common bean genotypes Arc.1, Arc.2, Arc.1S, Arc.3S, and Arc.5S were identified as resistant to *A. obtectus*. Some Bulgarian common bean genotypes and commonly grown Turkish bean genotypes were resistant against *A. obtectus* [42, 43]. Although an increasing number of common bean germplasms resistant to weevils have been discovered, most of these are concentrated in wild beans, and few are concentrated in cultivated beans.

At present, researches on bruchid resistance genes cloning are lagging behind both at home and abroad, and in particular, few studies have investigated on resistance genes in common bean cultivars using molecular markers. Blair et al. [44] used SSR molecular markers to identify a locus linked to arcelin 1 allele from the wild common bean accession RAZ106 and found that this locus was located at the APA locus on Pv04. Kamfwa et al. [45] mapped two QTLs on chromosome 4 (one associated with the APA locus) and one QTL on chromosome 6 using an *A. obtectus* resistant breeding line introgressed from the wild tepary bean (contained Arcelin gene). Lectin can be encoded by a single locus or by several loci, for example, APA locus located on Pv04, *Lec-2* and *Lec-3*, and the *lec* genes mapping on Pv07 [46, 47].

In this study, we found that the black kidney bean, a cultivar of common bean, has high resistance to *Acanthoscelides obtectus* (Say). We then constructed a high-density genetic linkage map using a recombinant inbred line population obtained from a cross between the bruchid-susceptible cultivar Longyundou3 and black kidney bean and performed gene mapping of bruchid resistance. This study mapped and analyzed the resistance genes for bean weevils in cultivated common bean, which may lay a foundation for further study on the mechanism of bruchid resistance in common bean and provide a possibility for improved seed preservation.

## Results

### Bruchid resistance segregation and genetic analysis

**Fig. 1:**
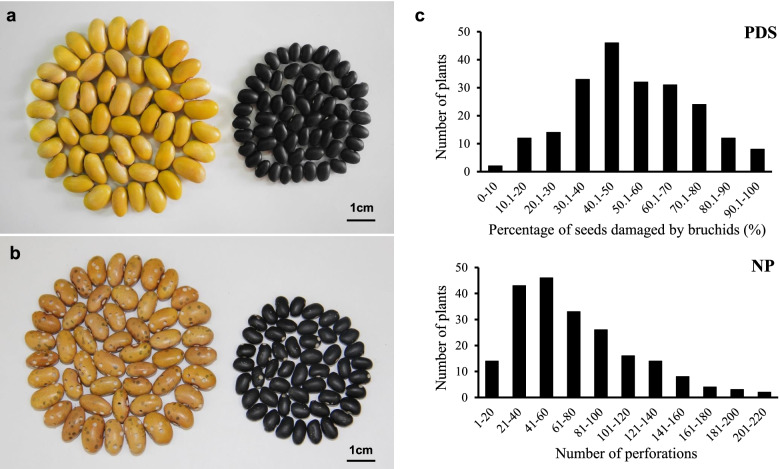
Phenotypic characteristics and frequency distribution of PDS and NP in the F_2:3_ families. The F_2:3_ families were derived from a cross between LYD3 (bruchid-susceptible parent) and BKB (bruchid-resistant parent). **a** Phenotypic characteristics of the two parents before infestation with bean weevils. **b** Phenotypic characteristics of the two parents after infestation with bean weevils. The large-yellow seeds on the left are the susceptible parent LYD3, and the small-black seeds on the right are from the resistant parent BKB. **c** Frequency distribution of the PDS and NP in the F_2:3_ families from a cross of LYD3 and BKB

The black kidney bean (BKB) exhibited resistance to *Acanthoscelides obtectus* (Say), with a mean percentage of damaged seeds of 28.3%. In contrast, Longyundou3 (LYD3) showed high susceptibility, and all the seeds were damaged by bruchids (percentage of damaged seeds equal to 100%) (Fig. 1a, b).

The resistance of F_2:3_ population and RILs was identified. Two traits, namely, the percentage of damaged seeds (PDS) and the number of perforations (NP), were investigated. The PDS among the F_2:3_ families ranged from 0 to 100%, and the NP ranged from 0 to 220, showing significant resistance segregation (Fig. 1c). The PDS in the RILs ranged from 0 to 100%, and the NP ranged from 0 to 280. However, the minimum damage rate of seeds was lower than that of resistant parent, which indicated that resistant trait had a positive superparent advantage. The distribution of bruchid resistance was continuous in the F_2:3_ population, and the ratio of susceptibility to resistance was not consistent with Mendelian inheritance. The kurtosis and skewness values were − 0.84 and 0.11, respectively, with the absolute values lower than one, which was approximately normal, and these results indicated the quantitative inheritance of the resistance trait controlled by polygenes.

### High-density genetic linkage map construction

Based on the technical characteristics of whole genome resequencing, the 157 RILs and two parents of common bean were resequenced. Raw reads were obtained by CASAVA base calling and conversion from the raw image data acquired by high-throughput sequencing. After filtering, 80,029,096 (17.12× coverage) and 71,913,622 (16.95× coverage) clean reads were generated from BKB (resistant parent) and LYD3 (susceptible parent), respectively. The clean reads from 157 RILs ranged from 39,686,552 to 105,924,272, with an average sequencing depth of 14.38× coverage. By comparing the sequencing data of BKB, LYD3 and 157 RILs with the reference genome, a total of 300,003 SNPs among the parents were screened out using GATK 3.8 software [48].

The sliding window approach, with 15 SNPs in each sliding window, was used in this study. According to the proportion of SNP markers in the sliding window from the male or female parents, the genotype of the segment in where the sliding window located was determined, and the location of the recombination breakpoint of the chromosome segment was determined. When the sliding window hit a homozygous/homozygous breakpoint, the genotype transiently changed from homozygous to heterozygous and then changed to the other homozygous genotype. A genotype remained unchanged until it hit a recombination breakpoint [49]. After all genotypes were called and the recombination breakpoints were determined, we identified a total of 48,689 recombinant breakpoints, with an average of 310 breakpoints per RIL (Fig. S1a).

**Fig. 2:**
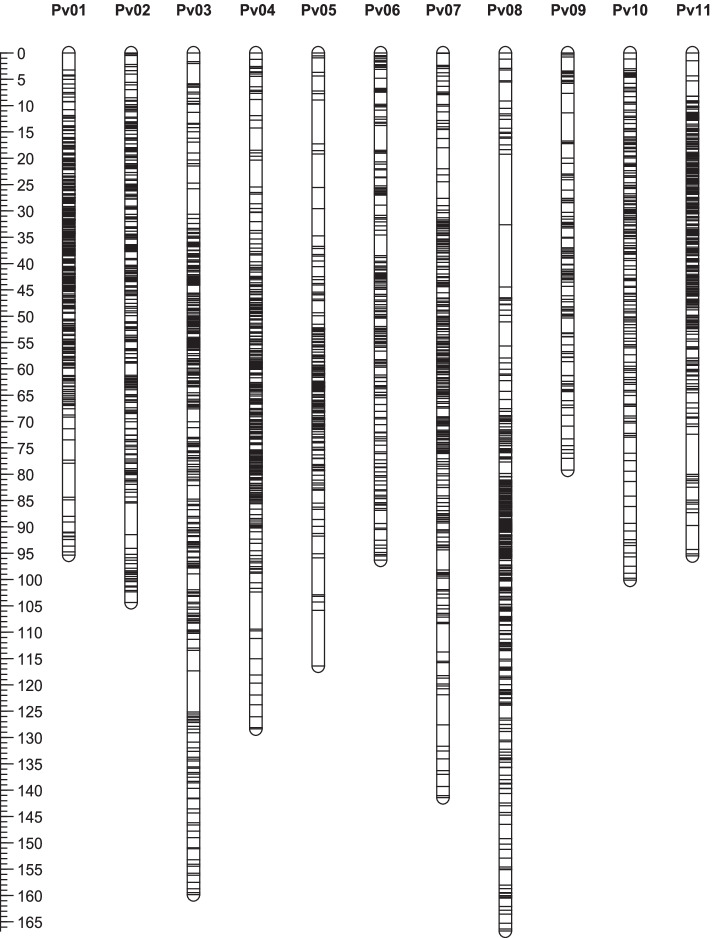
Genetic map of RILs from a cross between LYD3 and BKB. A high-density genetic map containing 11 chromosomes was constructed using 157 RILs derived from a cross between LYD3 (bruchid-susceptible parent) and BKB (bruchid-resistant parent). The bar on the left represents the genetic distance. The total genetic distance of the high-density map was 1283.68 cM, with an average interval of 0.61 cM between the bin markers. Chromosome 8 was the longest with a map length of 166.73 cM, contained 417 bin markers with 297,249 SNPs and had an average genetic distance of 0.71 cM. Chromosome 9 was the shortest, with a map length of 79.23 cM, which included 134 bin markers with an average genetic distance of 0.43 cM. The black lines represent the relative position of the bin markers on the genetic linkage group. More of the black lines indicate denser markers

By consolidating continuous non-recombination markers on the genome into a bin, a total of 4214 recombination bins were obtained for the 157 RILs. The average physical length of the recombination bins was 135.2 kb, including 712 SNPs. Each bin was used as a marker for genetic marker screening and genetic linkage map construction. Because segregation distortion could affect the results of map construction and QTL mapping, a ratio of 1:1 was used to perform segregation filtration with 4214 candidate markers, and 1108 biased markers were filtered out to yield 3106 effective markers. According to the genome information [50], the linkage groups were constructed using JoinMap 4.0 software [51] by controlling the LOD value between 2.0 and 10.0. A total of 11 linkage groups were generated with 3106 bin markers, containing 2,234,769 SNPs. Taking the linkage group as the unit, the genetic distance between the markers was calculated using the Kosambi algorithm. A high-density genetic map of a total map distance of 1283.68 cM, with an average interval of 0.61 cM between the bin markers, was constructed (Table 1, Fig. 2). The number of effective bin markers in each chromosome ranged from 134 to 417. The length of each chromosome ranged from 79.23 cM to 166.73 cM, and the average distance between bin markers ranged from 0.43 cM to 0.80 cM. With the exception of biggest gap > 10-cM on LG05 and LG08, all other biggest gaps were < 8-cM. The conversion ratio between genetic distance and physical distance ranged from 1.67 to 2.79. LG08 was the longest with a map length of 166.73 cM, containing 417 bin markers with 297,249 SNPs, and had an average genetic distance of 0.71 cM. LG03 was the second longest with a map length of 159.84 cM containing 289,659 SNPs. LG09 and LG01 were the two shortest with map lengths of 79.23 cM and 95.36 cM, including 134 bin markers with 110,344 SNPs and 364 bin markers with 246,412 SNPs, respectively, and the average genetic distances of LG09 and LG01 were 0.43 cM and 0.63 cM.

**Table 1:** Description of the basic characteristics of the genetic linkage map

LG ID^**a**^	Bin number^**b**^	Effective Bin Number^**c**^	SNP Number	Map length^**d**^ (cM)	Average distance^**e**^ (cM)	Max Gap^**f**^ (cM)	cM/Mb^**g**^
LG01	444	364	246,412	95.36	0.63	6.46	1.67
LG02	403	288	264,367	104.37	0.56	6.01	1.93
LG03	443	366	289,659	159.84	0.65	7.81	2.79
LG04	380	328	252,752	128.37	0.60	7.05	2.52
LG05	337	198	78,079	116.43	0.58	10.56	2.54
LG06	261	191	160,566	96.32	0.57	4.71	2.61
LG07	381	286	196,439	141.40	0.66	5.77	2.49
LG08	449	417	297,249	166.73	0.71	13.39	2.58
LG09	329	134	110,344	79.23	0.43	5.36	1.87
LG10	355	177	109,441	100.12	0.52	3.19	2.07
LG11	432	357	229,461	95.51	0.80	7.63	1.76
Total	4214	3106	2,234,769	1283.68	0.61	13.39	2.26

The high-density genetic linkage map in this study contained 11 linkage groups

^a^LG ID represents the number of linkage groups

^b^Bin number represents the total number of bin markers in each group

^c^Effective bin number represents the total number of bin markers after filtering out biased separation markers

^d^Map length represents genetic distance of a linkage group

^e^Average distance (cM) represents the average genetic distance of bin markers on each linkage group

^f^Max gap (cM) represents the biggest gap in each linkage group

^g^cM/Mb represents the conversion ratio between genetic distance and physical distance

Collinearity analysis was performed based on the positions of markers on the genome and the genetic map, and the results showed that the order of most marker positions on each linkage group was consistent with the chromosome (Fig. S1b), indicating good collinearity, high accuracy of the genetic recombination rate and high accuracy of the map construction.

### QTL analysis and mapping for bruchid resistance

**Fig. 3:**
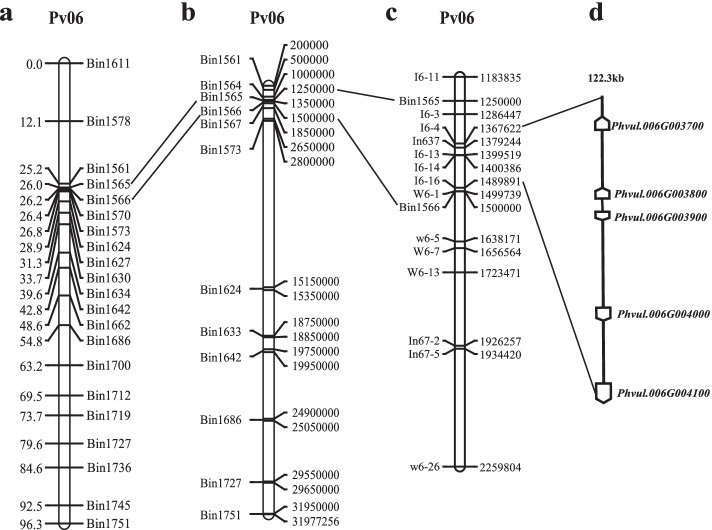
Genetic and physical maps of QTL loci for resistance to *Acanthoscelides obtectus* and candidate genes analysis. **a** Genetic map of the QTL for both PDS and NP in linkage group 6 using 157 RILs derived from LYD3 × BKB. The markers list at the right and left are the genetic positions (cM). **b** Physical map of the bin markers on chromosome 6. The markers list on the left and right are the physical positions in base pairs (bp). **c** Delimitation of the *qAO6.1* locus to a 122.3 kb region of chromosome 6. **d** Predicted candidate genes between markers I6–4 and I6–16 according to the physical position on the reference genome common bean accession G19833v1.0

The genetic map was used to identify QTLs controlling bruchid resistance in common bean. After the identification of bruchid resistance, the PDS of the RILs ranged from 0 to 100%, and the NP ranged from 0 to 280. There was a significant positive correlation between the two phenotypes, with a correlation coefficient of 0.88 (Fig. S2). The ICIM-ADD method in QTL IciMapping 4.2 software was used to analyse PDS and NP. The analysis of the 157 RILs detected one QTL associated with PDS located on chromosome 6 between markers Bin1565 and Bin1566. The LOD value was 5.49, explaining 16.10% of the phenotypic variation, and the additive effect was − 0.11. One QTL related to NP, which was also located on chromosome 6 between markers Bin1565 and Bin1566, had the LOD value of 4.31 and additive effect of − 27.48, and explained 16.37% of the phenotypic variation (Table 2; Fig. 3a; Fig. S3). This QTL for both PDS and NP between markers Bin1565 and Bin1566 was 0.23 cM, and located at the physical position between 1,250,000 and 1,500,000 bp on chromosome 6 (Fig. 3b). We named the QTL *qAO6.1*, and the beneficial alleles at this locus came from BKB, the resistant parent. The *qAO6.1* might be a major locus for resistance to *Acanthoscelides obtectus*.

**Table 2:** Location and effects of QTLs controlling resistance to *Acanthoscelides obtectus* detected in RILs

trait	QTL name	Chromosome	Marker interval^**a**^	Interval size (cM)	LOD^**b**^	PVE^**c**^ (%)	Add^**d**^
PDS	*qAO6.1*	6	Bin1565-Bin1566	0.23	5.49	16.10	−0.11
NP		6	Bin1565-Bin1566	0.23	4.31	16.37	−27.48

^a^The QTL was located between left and right markers

^b^The LOD threshold was determined using 1000 permutations

^c^PVE was the phenotypic variation explained by the QTL

^d^Add represents the additive effect

For narrowing down the region identified by QTL-seq, we developed 44 SSR markers and 5 InDel markers (Table S1) on chromosome 6 in candidate region to detect polymorphisms between BKB and LYD3. A total 11 SSR markers and 3 InDel markers on chromosome 6 were polymorphic between the two parents. The regional linkage map for chromosome 6 were constructed with polymorphic DNA markers using F_2_ mapping population consisted of 188 individual plants. We identified ten recombinants from the fine-mapping population using seven molecular markers. The *qAO6.1* locus was delimited to an interval of approximately 122.3 kb between SSR markers I6–4 and I6–16 (Fig. 3c).

To further verify the correlation between this locus and bruchid resistance, an association study in candidate region was conducted using SNP markers and the phenotypic data of two traits, PDS and NP, of one natural population (contained 628 common bean accessions) (Table S2). A total 437 SNPs were generated from the whole genome resequencing project between I6–4 (Pv06, 1,367,622 bp) and I6–16 (Pv06, 1,489,891 bp) on chromosome 6. The results showed that 70 SNPs (*P*-value < 1 × 10^− 6^) associated with PDS and 7 SNPs (*P*-value < 1 × 10^− 6^) associated with NP were detected (Table S3). Seven SNPs (Pv06_1413364, Pv06_1429130, Pv06_1429225, Pv06_1429229, Pv06_1429376, Pv06_1432127, Pv06_1,432,316) were associated with both two traits. The peak SNP (SNP with the lowest *P-*value) was located in 1,432,316 bp on chromosome 6 (PDS-associated Pv06_1,432,316, *P* = 8.38 × 10^− 28^; NP-associated Pv06_1,432,316, *P* = 3.44 × 10^− 12^) (Fig. S3). Four SNPs associated with PDS (Pv06_1376148, Pv06_1376347, Pv06_1376967 and Pv06_1407036) were located within genes, others located between genes. These results suggested that this locus might be strongly associated with bruchid resistance.

### Annotation and candidate gene prediction for candidate region

According to the common bean reference genome sequence of G19833v1.0, the region between markers I6–4 and I6–16 contained five genes, *Phvul.006G003700* (Pv06, 1,376,123–1,378,343 bp), *Phvul.006G003800* (Pv06, 1,406,616–1,408,098 bp), *Phvul.006G003900* (Pv06, 1,417,873–1,419,245 bp), *Phvul.006G004000* (Pv06, 1,454,577–1,457,697 bp) and *Phvul.006G004100* (Pv06, 1,486,729–1,491,368 bp) (Fig. 3d; Table 3). *Phvul.006G003700* was found to be a homologous gene in *A. thaliana* according to the NCBI database and encoded a bifunctional inhibitor/seed storage/LTP family protein, belonging to the AAI-LTSS [alpha-amylase inhibitor (AAI), lipid transfer (LT) and seed storage (SS)] protein superfamily. The AAI-LTSS family is known to play important roles in defending plants from insects and pathogens, in lipid transport between intracellular membranes, and in nutrient storage. The BLAST searches against the NCBI and UniProt databases revealed that Phvul.006G003700 showed the strongest similarity to an AAI domain-containing protein in *Phaseolus vulgaris* (kidney bean) (identity = 91.29%) and an AAI domain-containing protein in *Glycine max* (soybean) (E-value = 0.0, score = 1614, identity = 77.9%), and it is speculated that the protein encoded by *Phvul.006G003700* might have a similar function of inhibiting α-amylase activity. *Phvul.006G003800* encodes a carbohydrate-binding X8 domain superfamily protein. *Phvul.006G004000* and *Phvul.006G004100* encode homologues of yeast ADA2 2B and ADA2 2A, respectively. *Phvul.006G003900* has unknown function. Based on reference genome sequence of G19833v2.1 (*Phaseolus vulgaris* v2.1, DOE-JGI and USDA-NIFA, http://phytozome.jgi.doe.gov/), *Phvul.006G003700* was corresponding to the *Phvul.007G119178* on Pv07 (17,491,322-17,493,543 bp).

**Table 3:** Gene annotation of the candidate region based on reference genome G19833v1.0 on chromosome 6

Gene name	Region^**a**^ (bp)	Gene annotations
*Phvul.006G003700*	1,376,123–1,378,343	Bifunctional inhibitor/lipid-transfer protein/seed storage 2S albumin superfamily protein
*Phvul.006G003800*	1,406,616–1,408,098	Carbohydrate-binding X8 domain superfamily protein
*Phvul.006G003900*	1,417,873–1,419,245	–
*Phvul.006G004000*	1,454,577–1,457,697	homolog of yeast ADA2 2B
*Phvul.006G004100*	1,486,729–1,491,368	homolog of yeast ADA2 2A

Horizontal line indicates that no putative conserved domains have been detected

^a^Region represents the physical location of candidate gene in G19833v1.0 genome

### Sequence and expression analysis of candidate gene

The coding DNA sequences (CDS) of *Phvul.006G003700* in BKB and LYD3 were determined. The amplification of the candidate gene indicated that *Phvul.006G003700* had a length of 999 bp in cDNA in the resistant genotype BKB and encoded 332 amino acid residues. No difference was found between BKB and LYD3 (Fig. S5). The results from the search of the SMART database showed that the Phvul.006G003700 protein contained an AAI domain in the region from amino acids 55 to 329 (Fig. S6).

**Fig. 4:**
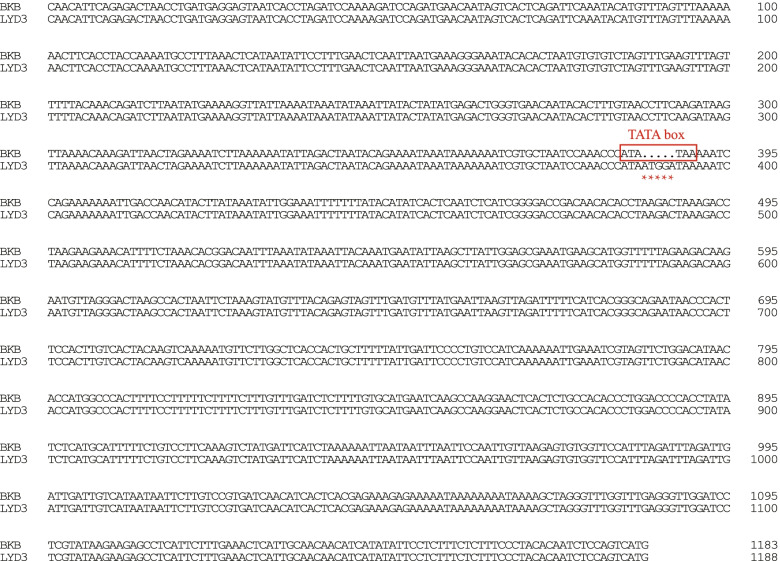
Sequence differences in promoter region of gene *Phvul.006G003700* between BKB (resistant) and LYD3 (susceptible). There was a 5 bp difference on 800 bp upstream of the start codon in the promoter region of gene *Phvul.006G003700* between LYD3 and BKB, locating on the essential *cis*-regulatory element TATA box of the promoter, resulting in the absence of TATA box in the susceptible parent. The TATA box was marked in red box. The red asterisks indicate the different base sites between two sequences

To further examine the transcriptional regulation of this gene, we analysed the upstream promoter sequence of the *Phvul.006G003700* gene using the PlantCARE database. The BLAST searches revealed that the amplified sequence included essential *cis*-regulatory elements of the promoter such as the core promoter element TATA and the common element CAAT that were highly conserved across the investigated species. We obtained the sequence of promoter region for *Phvul.006G003700* gene. A 5 bp difference on 800 bp upstream of the start codon in the promoter region of the *Phvul.006G003700* gene was found between LYD3 and BKB, which resulted in the absence of essential *cis*-regulatory element TATA box in the susceptible material (Fig. 4). TATA box was known to be an essential transcription regulator. The absence of the TATA box may reduce the efficiency of transcription and thus affect the gene expression of *Phvul.006G003700* in the susceptible parent LYD3.

**Fig. 5:**
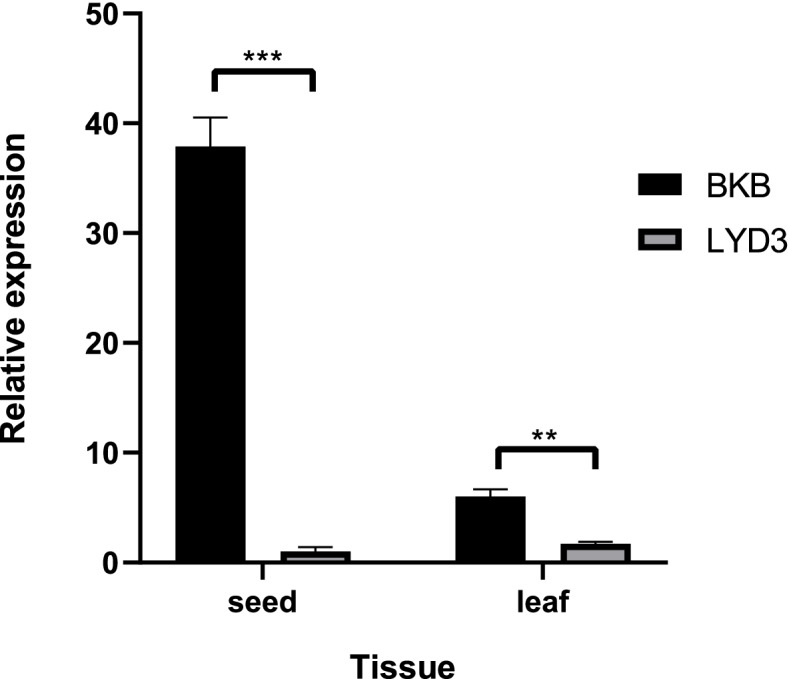
Relative quantitative expression analysis of *Phvul.006G003700* in seeds and leaves of BKB and LYD3 by qRT-PCR. The black bars represent BKB (bruchid-resistant parent), and the grey bars represent LYD3 (bruchid-susceptible parent). The horizontal axis of the graph indicates the different tissues, and the vertical axis indicates the relative expression levels. The data are presented as the means of three biological and technical replicates ± standard errors. ** indicates *P* < 0.01 and *** indicates *P* < 0.001 determined by a *t*-test

The qRT-PCR results showed that the expression level of *Phvul.006G003700* in BKB was markedly higher than LYD3 both in seeds and leaves (Fig. 5), which revealed that the gene expression level was higher in the bruchid-resistant accession than in the bruchid-susceptible accession. In seeds, gene *Phvul.006G003700* in BKB was about 37 times more expressed than it in LYD3, and about 3.5 times more expressed in leaves.

Through the results of association study between SNP markers and two traits of PDS and NP in the natural population, we found that three significant SNPs associated with PDS (Pv06_1376148, *P* = 5.85 × 10^− 10^; Pv06_1376347, *P* = 9.97 × 10^− 10^; Pv06_1376967, *P* = 1.11 × 10^− 9^) were localized in the 3′-UTR region of the candidate gene *Phvul.006G003700* (Fig. S7). These findings further support the likelihood that *Phvul.006G003700* was the candidate gene for bruchid resistance.

### Identification and homology sequence analysis of candidate gene

Homologous sequences were accessed of candidate gene *Phvul.006G003700* within the G19833 reference genome and other crops. We searched for homologous sequences of the candidate gene within the common bean G19833v1.0 reference genome and identified 14 additional specific genes located on chromosome 1, 3, 6, 8 and 9*.* Seven homologous genes, *Phvul.003G169100*, *Phvul.003G218600*, *Phvul.003G218700*, *Phvul.003G218800*, *Phvul.003G218900*, *Phvul.003G219000* and *Phvul.003G219100*, were located on chromosome 3. Four homologous genes, *Phvul.006G138600*, *Phvul.006G138800*, *Phvul.006G138900* and *Phvul.006G139100*, were located on chromosome 6. *Phvul.001G050300*, *Phvul.008G142500* and *Phvul.009G092000* were located on chromosome 1, 8 and 9, respectively. These homologous genes all had a similar protein domain as the candidate gene and encoded a bifunctional inhibitor/lipid-transfer protein/seed storage 2S albumin protein belonging to the AAI-LTSS protein superfamily. The analysis of conserved motifs revealed two motifs with an e-value >1E-60, and they were located within the AAI domain region of Phvul.006G003700 and homologous proteins in the *P. vulgaris* genome (Fig. S8).

To further clarify the relationship between the *Phvul.006G003700* gene and homologous genes from other crops, a BLAST search against the NCBI and UniProt databases was performed to find the homologous protein of Phvul.006G003700 in soybean (*Glycine max*), lupine (*Lupinus albus*), wheat (*Triticum aestivum*), maize (*Zea mays*), rice (*Oryza glumipatula*), barley (*Hordeum vulgare subsp. vulgare*), *Medicago truncatula*, *Arabidopsis thaliana* and other crops. The protein sequence encoded by *Phvul.006G003700* was compared with the homologous protein sequences in other crops by phylogenetic tree analysis (Fig. S9). The results showed that the Phvul.006G003700 protein was closely related to the AAI protein in soybean, which suggested that it might have a similar function to AAI. The analysis of conserved motifs revealed three motifs with an e-value >1E-60 among Phvul.006G003700 and homologous proteins in other crops, and the first two conserved motifs were located within the AAI domain region (Fig. S10).

## Discussion

During the storage of common bean, the damage caused by the common bean weevils leads to a decrease in stored grain [16, 17]. Although some physical and chemical methods [23, 24, 52] can be used to prevent and control the damage, the treated seeds are difficult to eat or sell again, resulting in relatively large economic losses [20]. The screening of resistant materials and identification of resistance genes of common bean are needed to provide a green and environmental protection approach. Previous studies have found some common bean germplasms that are resistant to bean weevils. Most resistant germplasms were concentrated in wild bean, and few were concentrated in cultivars/landraces. Based on the preliminary screening and identification of resistance to *A. obtectus* among several hundred common bean germplasm resources, we identified one cultivated common bean variety with good resistance to weevils. It was named black kidney bean (BKB), and presented an average seeds infestation rate of less than 40%. Using the cross between BKB and LYD3, a cultivar completely susceptible to *A. obtectus*, we constructed a segregated population. Through the identification of resistance to *A. obtectus* among the F_2:3_ population, we demonstrated that the trait of bruchid resistance was a quantitative trait.

The genetic linkage map is a powerful tool for gene mapping and cloning and is generally constructed using various molecular markers, such as SSR and SNP [53]. In our study, a high-throughput method for genotyping recombinant populations utilizing whole-genome resequencing data [54–57] was implemented, and a sliding window approach was designed to detect genome-wide SNPs for genotype calling and recombination breakpoint determination. This method is efficient and fast, and widely used in r crops, such as wheat and cucumber [49, 58]. We constructed a high-density genetic linkage map including 3106 bin markers using 157 common bean RILs derived from a cross between BKB and LYD3.The map contained 2,234,769 SNPs, and the number of markers were more than those in previous studies [59–61]. Compared to using F_2_ mapping population, using an RIL as the mapping population in our study can effectively reduce the influence of the dominant effect and reveal the additive effect of QTLs [62].

The researches on bruchid resistance genes in common bean are relatively few at present, and most of the genes associated with bruchid resistance exist in wild species, such as *Arcelin1* to *Arcelin8* [34, 36, 39, 40, 63–66]. In this study, we detected one QTL associated with both two traits, PDS and NP. This QTL mapped to the region on chromosome 6 between markers Bin1565 and Bin1566 and had the additive effects of − 27.48 and − 0.11, explaining 16.37% and 16.10% of the phenotypic variation, respectively. The additive effects were negative, indicating the beneficial alleles at this locus came from BKB, the resistant parent. We delimited this QTL to an interval of approximately 122.3 kb between SSR markers I6–4 and I6–16, and it may be a major locus for resistance to *Acanthoscelides obtectus*. This QTL was same located on chromosome 6, but approximately a distance of 22.9 Mb upstream of the QTL reported by Kamfwa [45], indicating that the *qAO6.1* identified in this study was a new locus related to bruchid resistance.

The *Phvul.006G003700* encodes a bifunctional inhibitor/seed storage/LTP family protein belonged to the AAI-LTSS protein superfamily, which is known to play important roles in defending plants from insects and pathogens, in lipid transport between intracellular membranes and in nutrient storage. The results from the search of the SMART database (https://smart.embl.de) [67] showed that the Phvul.006G003700 protein contained an AAI domain. Previous studies have been reported that a double-headed inhibitor (subtilis protease/α-amylase inhibitor and trypsin/α-amylase inhibitor) in Ragi (finger millet, *E. coracana* Gaertneri) could simultaneously bind a molecular protease (subtilis protease and trypsin) and a molecular α-amylase [68–70]. The α-amylase was a kind of important digestive enzymes in the saliva and gut of insects [71, 72], and proteinase inhibitors can combine with digestive enzymes to prevent insects from eating [73]. For example, proteinaceous inhibitor from finger millet could inhibit α-amylase of insect pests, and the highest inhibition percentage was found against α-amylase of *Callosobruchus chinensis* [74, 75]. The α-amylase inhibitor is an important biochemical substance in common bean, and plays an important role in insect resistance. The previous studies demonstrated that the seeds of adzuki bean with a transferred α-amylase inhibitor gene could accumulate a high amount of α-amylase inhibitor and had a high resistance to bruchid [27]. Similarly, one transgenic chickpea and four cowpea lines expressing αAI-1 (an α-amylase inhibitor from common bean) were highly resistant to both bruchid pest species *Callosobruchus chinensis* and *Callosobruchus maculatus* [76]. Franco et al. [77, 78] confirmed plant α-amylase inhibitors can inhibit the digestive enzymes from the bean weevil *Acanthoscelides obtectus*. According to the analysis of candidate gene sequences, we found that there was a 5 bp difference in the promoter region of *Phvul.006G003700* between the two parents, which was just located in the TATA box region. TATA box is an essential transcription regulator and may affect gene expression level. The qRT-PCR analysis of gene *Phvul.006G003700* showed that the expression level of *Phvul.006G003700* in bruchid-resistant parent was much higher than that in bruchid-susceptible parent in dry seeds, which corresponded to the presence of TATA box in resistant parent and the absence of TATA box in susceptible parent due to 5 bp insertion. Basi´ studies [79] showed that the stepwise deletions of the TATA box of *nmt1* gene highly expressed in *Schizosaccharomyces pombe* could result in the decrease in transcription efficiency and level of expression down-regulated. Therefore, we speculated that the high level of *Phvul.006G003700* gene expression in BKB could lead to the high content of α-amylase inhibitor, thus enhancing the inhibition to α-amylase of *Acanthoscelides obtectus*, and producing anti-insect effect. Further studies for the functional validation of this candidate gene are required, for example using complementation or gene knockout analyses, and physiological and biochemical researches.

## Conclusions

We constructed a high-density genetic map by whole-genome resequencing using a RIL population. The map has 3106 bin markers, containing 2,234,769 SNPs. A new major QTL for resistance to *A. obtectus* on chromosome 6 was identified from a common bean cultivar based on the constructed genetic map. *Phvul.006G003700* encodes a bifunctional α-amylase/protease-inhibited protein and is likely to be a candidate gene responsible for bruchid resistance. This research was very useful for screening and identifying bruchid resistance resources in common bean, and further research on the excavation of bruchid-resistant genes.

## Methods

### Plant materials

The black kidney bean (BKB), a Chinese cultivar in the Mesoamerican gene pool of common bean that has a determinate bush plant and small black seeds (100-seed weight of 17.05 g ± 1.38), displays bruchid resistance. Longyundou3 (LYD3), a Chinese cultivar originating from the Andean gene pool, is an indeterminate viny plant with large yellow seeds (100-seed weight of 59.23 g ± 4.00) and susceptible to *Acanthoscelides obtectus* (Say). A genetic mapping population was created from a cross between LYD3 and BKB, and the F_6_ population, which included 157 recombinant inbred lines (RILs), was obtained after continuous self-crossing. All seeds production was carried out at the experimental field of Heilongjiang Academy of Agricultural Sciences at the Harbin and Changping experimental sites of the Chinese Academy of Agricultural Sciences in Beijing, China.

### Evaluation of bruchid resistance

The improved indoor artificial pest inoculation method was used to identify the resistance to *Acanthoscelides obtectus*. *Acanthoscelides obtectus* was collected from local red kidney beans infested with partially feathered bean weevils located in several cities and counties with severe bruchid damage in Yunnan Province. Forty healthy dry seeds were randomly selected from harvested seeds of each F_2_ individual and F_6_ line. Trials were replicated two times, with 20 seeds per replicate. Fifty healthy dry seeds of resistant parent and susceptible parent were used as control. Each material was placed into a 9-cm-diameter petri dish and randomly placed on the shelves. We covered all the shelves with a fine net, and put thousands of the collected feathered adults of *A. obtectus* (male and female) into the net to mate freely, so as to ensure that all the seeds were infested under the same condition. The room temperature was maintained at 18 ~ 25 °C. The humidity was maintained at approximately 70%, and timely ventilation was performed. After 100 days, the damage degree of each material was investigated. The percentage of damaged seeds (PDS) and the number of perforations (NP) were calculated. The percentage of damaged seeds was the percentage of infested seeds in the total number of identified seeds, and the number of perforations was the total number of infested holes of all identified seeds in each material.

### Genetic map construction of the RIL population

BKB, LYD3 and 157 RILs from a cross between BKB and LYD3 were used for resequencing. The DNA samples of sufficient quality were randomly interrupted into 350-bp fragments using a Bioruptor Pico ultrasonic crushing machine, and a genomic DNA library construction kit was used for library preparation, including DNA fragment end flatting, adding an ‘A’ to 3′ end, connecting an adaptor with DNA end and PCR amplification. Quality control was performed with the constructed libraries. The libraries of sufficient quality were sequenced on an Illumina HiSeq2500 for high-throughput sequencing, generally using PE150 sequencing mode, that is, double-end sequencing with 150 bp measured at each end. The raw image data files obtained by high-throughput sequencing were analysed by CASAVA base calling (http://support.illumina.com/sequencing/sequencing_software/casava.ilmn) [80] and converted into original sequencing sequences, which were called raw data or raw reads. After filtering, clean and effective reads were obtained. The clean reads of the two parents and 157 RILs were compared with the reference genome G19833v1.0 [50] using BWA software [81]. The single nucleotide polymorphisms (SNPs) of each sample were detected using GATK 3.8 software [48], and tens of thousands of SNP markers were developed.

The SNPs were filtered based on the missing percentage ≤ 20% and MAF ≥ 5%. We combined the SNP markers that were continuously unrecombined in the genome into a bin. First, the sliding window method was used to construct a recombination map. Each sliding window contained 15 SNPs. According to the proportion of SNP markers in the sliding window from the male or female parents, the genotype of the segment in where the sliding window located was determined, and the location of the recombination breakpoint of the chromosome fragment was identified. A recombination map was then drawn. Second, a bin map was constructed based on the recombination map of all the families in the group. Each bin was used as a marker for genetic marker screening and genetic linkage map construction. Taking the linkage group as a unit, JoinMap 4.0 software [51] was used to construct the genetic linkage map, and the Kosambi algorithm was used to calculate the genetic distance between markers.

### QTL mapping for bruchid resistance

Quantitative trait loci (QTLs) were identified by inclusive composite interval mapping (ICIM) using QTL IciMapping4.2 software [82]. A 1-cM scan window was employed, and the likelihood ratio statistic was computed every 2 cM. The LOD (log of odds) values and PVE were determined based on likelihood ratio tests under a hypothesis allowing both additive and dominance effects. A 1000 permutation test was used, and QTLs with LOD values of 3.0 and higher were called for candidate loci.

### DNA markers development

The candidate region for bruchid resistance was verified using the analysis of SSR (simple sequence repeats) markers and InDel (insertion-deletion) markers. The SSR markers and InDel markers were designed in primer5 (Premier Biosoft International, USA). According to the location of candidate intervals in the reference genome G19833v1.0 [50], forty-four new SSR markers and 5 InDel markers on chromosome 6 were developed to screen for polymorphisms between BKB and LYD3 (Table S1). Polymerase chain reaction (PCR) was performed in 10 μL reactions containing 20–25 ng of template DNA, 0.2 μmol L^− 1^ forward and reverse primers each 0.5 μL, 5 μL of 2 × Taq DNA polymerase Mix and suitable nuclease-free water in a T100 Thermal Cycler (Bio-Rad Research, USA). The amplification profile was followed by 95 °C for 5 min, 30 cycles of 95 °C for 30 s, 50 °C–60 °C for 30s, 72 °C for 1 min/1 kb, and final extension for 10 min at 72 °C. The amplified products were electrophoresed on 8% nondenaturing polyacrylamide gels, and the bands were visualized by using silver nitrate staining.

### Association study of candidate region

One natural population (a total of 628 common bean accessions representing broad genetic diversity, including 504 accessions in China and 124 elsewhere) was used in the association study for candidate region (Table S2). Genotypic data were generated from the whole genome resequencing project of 683 accessions as previously described by Wu [1], and nucleotide variations were further filtered based on the missing rates (≤ 10%) and a minor allele frequency of ≥ 0.05. Two traits of 628 common bean accessions, PDS and NP, were assessed in this study. The general linear model (GLM) [83] was used to detect the association between markers and phenotypes using Tassel 5.0. The *P-*value threshold was 1 × 10^− 6^ [2], and the markers that exceeded the threshold (1 × 10^− 6^) were considered to be associated with PDS or NP.

### Annotation of candidate genes

The genes of candidate regions were annotated using the annotation file of common bean accession G19833v1.0 [50]. The homologous genes of common bean candidate genes were searched in *Arabidopsis thaliana* and other crops according to the NCBI (https://www.ncbi.nlm.nih.gov/) and UniProt (https://www.uniprot.org/) databases.

### Sequence amplification of candidate genes for bruchid resistance

Based on the results from QTL mapping, gene-specific primer pairs (Table S4) were developed and used to amplify the genomic DNA (gDNA), complementary DNA (cDNA) and promoter sequences of the candidate genes in BKB and LYD3. PCR amplification was performed in 50 μL volumes containing 100 ng of template DNA, 1 μL of each forward and reverse primer (10 μmol L^− 1^), 25 μL of 2 × Trans Start® KD Plus Super Mix (−dye) and the suitable nuclease-free water in a T100 Thermal Cycler (Bio-Rad Research, USA). The thermal cycling programme was as follows: 94 °C for 5 min, 35 cycles of 94 °C for 30 s, 53–60 °C for 30 s, and 68 °C for 1 kb/min, and final extension at 72 °C for 8 min. The amplified products were electrophoresed on a 1.0% agarose gel. The target bands were cut and purified using a TIANgel Midi Purification Kit, then connected with the *pEASY*®-Blunt Cloning vector and transferred into the newly thawed *Trans*1-T1 Phage Resistant Chemically Competent Cell. The connected product was coated on an LB plate containing IPTG, X-gal and ampicillin antibiotics and cultured overnight. White colonies were selected for detection and sequencing.

### Bioinformatics analysis

Sequence alignment was performed by MEGA 6.0 software [84]. The domains of the proteins encoded by the candidate genes were identified and annotated using a search of the SMART (Simple Modular Architecture Research Tool) database (https://smart.embl.de) [67]. A BLAST search against the NCBI and UniProt databases was performed to find homologous proteins from the common bean G19833 reference genome (*Phaseolus vulgaris*), soybean (*Glycine max*), wild soybean (*Glycine soja*), lupine (*Lupinus albus*), wheat (*Triticum aestivum*), maize (*Zea mays*), rice (*Oryza glumipatula*), barley (*Hordeum vulgare subsp. vulgare*), *Medicago truncatula*, *Arabidopsis thaliana* and others. Phylogenetic analysis of the candidate genes from common bean was performed by comparison with homologous sequences from other crops. Phylogenetic trees were constructed using the neighbour-joining method as implemented in MEGA 6.0 software with 1000 bootstrap replicates [85]. MEME software [86] was used to identify the motifs of conserved sequences.

### Candidate gene expression analysis by qRT-PCR

We selected dry seeds and fresh leaves of BKB and LYD3 for quantitative real-time PCR (qRT-PCR) detection. Total RNA was extracted from the seeds using an improved method combining the cold phenol method [87] and trizol reagent [88], and reverse transcribed into cDNA using a reverse transcription kit (Tiangen, Beijing, China). qPCR was performed in a 20 μL volume consisting of 2 μL of cDNA (1 ng), 0.4 μL of each gene-specific forward and reverse primer (10 μM) (Table S5), 10 μL of 2 × *TransStart*® Top Green qPCR Super Mix (+dyeII) and a suitable amount of nuclease-free water. The reaction was run on an ABI 7500 real-time PCR machine (Bio-Rad Corporation, USA) using the following programme: 94 °C for 30 s, 40 cycles of 94 °C for 5 s and 56 °C for 15 s and 72 °C for 10 s. The *Actin* gene of common bean was used as a reference gene [89]. The 2^−ΔΔCT^ method [90] was used to analyse the gene expression data from three biological replicates and three technical replicates.

## Supplementary Information

**Additional file 1.**

**Additional file 2.** 

## Data Availability

The data supporting the findings of this study are available from the corresponding author (Jing Wu) upon request.

## Abbreviations

APA: Arcelin, phytohemagglutinin, and a-amylase
LOD: Logarithm of odds
QTL: Quantitative trait loci
RIL: Recombinant inbred line
LG: Linage group
SNP: Single nucleotide polymorphism
SSR: Simple sequence repeats
InDel: Insertion-deletion
PDS: The percentage of damaged seeds
NP: The number of perforations
αAI/AAI: alpha-amylase inhibitor
qRT-PCR: Quantitative real-time reverse transcription-polymerase chain reaction

## References

[1] Wu J, Wang L, Fu J, Chen J, Wei S, Zhang S (2020). Resequencing of 683 common bean genotypes identifies yield component trait associations across a north–south cline. Nat Genet.

[2] Wu L, Chang Y, Wang L, Wu J, Wang S (2021). Genetic dissection of drought resistance based on root traits at the bud stage in common bean. Theor Appl Genet.

[3] Bitocchi E, Rau D, Bellucci E, Rodriguez M, Murgia ML, Gioia T, Santo D, Nanni L, Attene G, Papa R (2017). Beans (*Phaseolus* ssp.) as a model for understanding crop evolution. Front Plant Sci.

[4] Myers JR, Kmiecik K, Pérez de la Vega M, Santalla M, Marsolais F (2017). Common bean: economic importance and relevance to biological science research. The common bean genome.

[5] Broughton WJ, Hernández G, Blair M, Beebe S, Gepts P, Vanderleyden J (2003). Beans (*Phaseolus* spp.) – model food legumes. Plant and Soil.

[6] Parreira JR, Bouraada J, Fitzpatrick MA, Silvestre S, de Silva AB, da Silva JM, Almeida AM, Fevereiro P, Altelaar AFM, Araújo SS (2016). Differential proteomics reveals the hallmarks of seed development in common bean (*Phaseolus vulgaris* L.). J Proteomics.

[7] Mensack MM, Fitzgerald VK, Ryan EP, Lewis MR, Thompson HJ, Brick MA (2010). Evaluation of diversity among common beans (*Phaseolus vulgaris* L.) from two centers of domestication using 'omics' technologies. BMC Genomics.

[8] Zhang X, Blair MW, Wang S (2008). Genetic diversity of Chinese common bean (*Phaseolus vulgaris* L.) landraces assessed with simple sequence repeat markers. Theor Appl Genet.

[9] Toledo MEO, de Mejia EG, Sivaguru M, Amaya-Llano SL (2016). Common bean (*Phaseolus vulgaris* L.) protein-derived peptides increased insulin secretion, inhibited lipid accumulation, increased glucose uptake and reduced the phosphatase and tensin homologue activation in vitro. J Funct Foods.

[10] Naroz MH, Ahmed SS, Abdel-Aziz SY, Abdel-Shafy S (2019). First record of *Acanthoscelides obtectus* (say) (Coleoptera: Chrysomelidae: Bruchinae) in Egypt: development and host preference on five species of legume seeds. Coleopt Bull.

[11] Mallqui KSV, Oliveira EE, Guedes RNC (2013). Competition between the bean weevils *Acanthoscelides obtectus* and *Zabrotes subfasciatus* in common beans. J Stored Prod Res.

[12] Alvarez N, McKey D, Hossaert-Mckey M, Born C, Mercier L, Benrey B (2005). Ancient and recent evolutionary history of the bruchid beetle, *Acanthoscelides obtectus* say, a cosmopolitan pest of beans. Mol Ecol.

[13] González-Vélez A, Ferwerda F, Abreu E, Beaver JS (2012). Development of bean lines (*Phaseolus vulgaris* L.) resistant to BGYMV, BCMNV and bean weevil (*Acanthoselides obtectus* say). Annu Rep Bean Improv Coop.

[14] Simmonds MSJ, Blaney WM, Birch ANE (1989). Legume seeds: the defences of wild and cultivated species of *Phaseolus* against attack by bruchid beetles. Ann Bot.

[15] Duan C, Zhu Z, Li W, Bao S, Wang X (2017). Genetic diversity and differentiation of *Acanthoscelides obtectus* say (Coleoptera: Bruchidae) populations in China. Agric For Entomol.

[16] Mutungi C, Affognon HD, Njoroge AW, Manono J, Baributsa D, Murdock LL (2015). Triple-layer plastic bags protect dry common beans (*Phaseolus vulgaris*) against damage by *Acanthoscelides obtectus* (Coleoptera: Chrysomelidae) during storage. J Econ Entomol.

[17] Oliveira MRC, Corrêa AS, de Souza GA, Guedes RNC, de Oliveira LO (2013). Mesoamerican origin and pre- and post-columbian expansions of the ranges of *Acanthoscelides obtectus* say, a cosmopolitan insect pest of the common bean. PLoS One.

[18] Johnson JA, Valero KA (2000). Control of cowpea weevil, *Callosobruchius maculatus*, using freezing temperatures. Annual International Research Conference on Methyl Bromide Alternatives and Emissions Reduction.

[19] Ferizli AG, Emekci M, Tutuncu S, Navarro S (2004). Utilization of freezing temperatures to control *Callosobruchus maculatus* fab. (Coleoptera, Bruchidae). Integrated Protection of Stored Products.

[20] Manickam L, Jayas DS, Fields PG, White NDG (2011). Low and high temperatures for the control of cowpea beetle, *Callosobruchus maculatus* (F.) (coleoptera: Bruchidae) in chickpeas. J Stored Prod Res.

[21] Agrafioti P, Athanassiou GG, Nayak MK (2019). Detection of phosphine resistance in major stored-product insects in Greece and evaluation of a field resistance test kit. J Stored Prod Res.

[22] Ren Y, Mahon D (2006). Fumigation trials on the application of ethyl formate to wheat, split faba beans and sorghum in small metal bins. J Stored Prod Res.

[23] Mushobozy DMK, Nganilevanu G, Ruheza S, Swella GB (2009). Plant oils as common bean (*Phaseolus vulgaris* L.) seed protectants against infestations by the Mexican bean weevil *Zabrotes subfasciatus* (Boh.). J Stored Prod Res.

[24] Negrisoli CRCB, Júnior ASN, Bernardi D, Garcia MS (2013). Activity of eight strains of entomopathogenic nematodes (Rhabditida: Steinernematidae, Heterorhabditidae) against five stored product pests. Exp Parasitol.

[25] Schmale I, Wackers FL, Cardona C, Dorn S (2006). Biological control of the bean weevil, *Acanthoscelides obtectus* (say) (Col.: Bruchidae), by the native parasitoid *Dinarmus basalis* (Rondani) (Hym.: Pteromalidae) on small-scale farms in Colombia. J Stored Prod Res.

[26] Ballhorn DJ, Kautz S, Heil M (2013). Distance and sex determine host plant choice by herbivorous beetles. PLoS One.

[27] Ishimoto M, Sato T, Chrispeels MJ, Kitamura K (1996). Bruchid resistance of transgenic azuki bean expressing seed α-amylase inhibitor of common bean. Entomol Exp Appl.

[28] Lioi L, Galasso I, Lanave C, Daminati MG, Bollini R, Sparvoli F (2007). Evolutionary analysis of the APA genes in the *Phaseolus* genus: wild and cultivated bean species as sources of lectin-related resistance factors?. Theor Appl Genet.

[29] Kami J, Poncet V, Geffroy V, Gepts P (2006). Development of four phylogenetically-arrayed BAC libraries and sequence of the APA locus in *Phaseolus vulgaris*. Theor Appl Genet.

[30] Mbogo KP, Davis J, Myers JR (2009). Transfer of the arcelin-phytohaemagglutinin-α amylase inhibitor seed protein locus from tepary bean *(Phaseolus acutifolius* a. gray) to common bean (*P. vulgaris* L.). Biotechnology..

[31] Paes NS, Gerhardt IR, Coutinho MV, Yokoyama M, Santana E, Harris N, Chrispeels MJ, Grossi-de-Sá MF (2000). The effect of arcelin-1 on the structure of the midgut of bruchid larvae and immunolocalization of the arcelin protein. J Insect Physiol.

[32] Santino A, Valsasina B, Lioi L, Vitale A, Bollini R (1991). Bean (*Phaseolus vulgaris* L.) seed lectins: a novel electrophoretic variant of arcelin. Plant Physiol.

[33] Velten G, Rott AS, Cardona C, Dorn S (2007). The inhibitory effect of the natural seed storage protein arcelin on the development of *Acanthoscelides obtectus*. J Stored Prod Res.

[34] Lioi L, Sparvoli F, Galasso I, Lanave C, Bollini R (2003). Lectin-related resistance factors against bruchids evolved through a number of duplication events. Theor Appl Genet.

[35] Osborn TC, Alexander DC, Sun SSM, Cardona C, Bliss FA (1988). Insecticidal activity and lectin homology of Arcelin seed protein. Science..

[36] Andreas JR, Yandell BS, Bliss FA (1986). Bean arcelin 1. Inheritance of a novel seed protein of *Phaseolus vulgaris* L. and its effect on seed composition. Theor Appl Genet.

[37] Goossens A, Quintero C, Dillen W, Rycke RD, Valor JF, Clercq JD, Van Montagu M, Cardona C, Angenon G (2000). Analysis of bruchid resistance in the wild common bean accession G02771: no evidence for insecticidal activity of arcelin 5. J Exp Bot.

[38] Blair MW, Prieto S, Díaz LM, Buendía HF, Cardona C (2010). Linkage disequilibrium at the APA insecticidal seed protein locus of common bean (*Phaseolus vulgaris* L.). BMC Plant Biol.

[39] Acosta-Gallegos JA, Quintero C, Vargas J, Toro O, Tohme J, Cardona C (1998). A new variant of arcelin in wild common bean, *Phaseolus vulgaris* L., from southern Mexico. Genet Resour Crop Evol.

[40] Zaugg I, Magni C, Panzeri D, Daminati MG, Bollini R, Benrey B, Bacher S, Sparvoli F (2013). QUES, a new *Phaseolus vulgaris* genotype resistant to common bean weevils, contains the Arcelin-8 allele coding for new lectin-related variants. Theor Appl Genet.

[41] Baldin ELL, Lara FM, Camargo RS, Pannuti LER (2017). Characterization of resistance to the bean weevil *Acanthoscelides obtectus* say, 1831 (Coleoptera: Bruchidae) in common bean genotypes. Arthropod Plant Interact.

[42] Apostolova E, Palagacheva N, Svetleva D, Mateeva A (2013). Investigations on the resistance of some Bulgarian common bean genotypes towards bean weevil (*Acanthoscelides obtectus* say). J Cent Eur Agric.

[43] Azizoglu U (2018). Biochemical properties of Turkish common beans and their resistance against bean weevil *Acanthoscelides obtectus* (Coleoptera: Bruchidae). Arthropod Plant Interact.

[44] Blair MW, Muñoz C, Buendía HF, Flower J, Bueno JM, Cardona C (2010). Genetic mapping of microsatellite markers around the arcelin bruchid resistance locus in common bean. Theor Appl Genet.

[45] Kamfwa K, Beaver JS, Cichy KA, Kelly JD (2018). QTL mapping of resistance to bean weevil in common bean. Crop Sci.

[46] Duarte MAG, Cabral GB, Ibrahim AB, Aragão FJL (2018). An overview of the APA locus and arcelin proteins and their biotechnological potential in the control of bruchids. Agri Gene.

[47] Joshi J, Pandurangan S, Diapari M, Marsolais F, Pérez dela Vega M, Santalla M, Marsolais F (2017). Comparison of gene families: seed storage and other seed proteins. The common bean genome.

[48] DePristo MA, Banks E, Poplin R, Garimella KV, Maguire JR, Hartl C (2011). A framework for variation discovery and genotyping using next-generation DNA sequencing data. Nat Genet.

[49] Huang X, Feng Q, Qian Q, Zhao Q, Wang L, Wang A (2009). High-throughput genotyping by whole-genome resequencing. Genome Res.

[50] Schmutz J, McClean PE, Mamidi S, Wu GA, Cannon SB, Grimwood J (2014). A reference genome for common bean and genome-wide analysis of dual domestications. Nat Genet.

[51] Van Ooijen JW (2006). JoinMap® 4.0: software for the calculation of genetic linkage maps in experimental population.

[52] Baier A, Webster BD (1992). Control of *Acanthoscelides obtectus* say (Coleoptera: Bruchidae) in *Phaseolus vulgaris* L. seed stored on small farms—I. evaluation of damage. J Stored Prod Res.

[53] Zheng X, Kuang Y, Zhang X, Lu C, Cao D, Li C, Sun X (2011). A genetic linkage map and comparative genome analysis of common carp (*Cyprinus carpio* L.) using microsatellites and SNPs. Mol Genet Genomics.

[54] Purcell S, Neale B, Todd-Brown K, Thomas L, Ferreira MAR, Bender D (2007). PLINK: a tool set for whole-genome association and population-based linkage analyses. Am J Hum Genet.

[55] Reuter JA, Spacek DV, Snyder MP (2015). High-throughput sequencing technologies. Mol Cell.

[56] Wang K, Li M, Hakonarson H (2010). ANNOVAR: functional annotation of genetic variants from high-throughput sequencing data. Nucleic Acids Res.

[57] Zhao Q, Huang X, Lin Z, Han B (2010). SEG-map: a novel software for genotype calling and genetic map construction from next-generation sequencing. Rice..

[58] Zhou Q, Miao H, Li S, Zhang S, Wang Y, Weng Y, Zhang Z, Huang S, Gu X (2015). A sequencing-based linkage map of cucumber. Mol Plant.

[59] Blair MW, Pedraza F, Buendia HF, Gaitán-Solís E, Beebe SE, Gepts P, Tohme J (2003). Development of a genome-wide anchored microsatellite map for common bean (*Phaseolus vulgaris* L.). Theor Appl Genet.

[60] Blair MW, Cortés AJ, Farmer AD, Huang W, Ambachew D, Penmetsa RV, Carrasquilla-Garcia N, Assefa T, Cannon SB (2018). Uneven recombination rate and linkage disequilibrium across a reference SNP map for common bean (*Phaseolus vulgaris* L.). PLoS One.

[61] Galeano CH, Cortés AJ, Fernández AC, Soler Á, Franco-Herrera N, Makunde G, Vanderleyden J, Blair MW (2012). Gene-based single nucleotide polymorphism markers for genetic and association mapping in common bean. BMC Genet.

[62] Liu C, Fan B, Cao Z, Su Q, Wang Y, Zhang Z, Tian J (2016). Development of a high-density genetic linkage map and identification of flowering time QTLs in adzuki bean (*Vigna angularis*). Sci Rep.

[63] Hartweck LM, Vogelzang RD, Osborn TC (1991). Characterization and comparison of arcelin seed protein variants from common bean. Plant Physiol.

[64] John ME, Long MC (1990). Sequence analysis of arcelin 2, a lectin-like plant protein. Gene..

[65] Goossens A, Geremia R, Bauw G, van Montagu M, Angenon G (1994). Isolation and characterization of arcelin-5 proteins and cDNAs. Eur J Biochem.

[66] Sparvoli F, Bollini R (1998). Arcelin in wild bean (*Phaseolus vulgaris* L.) seeds: sequence of arcelin 6 shows it is a member of the arcelins 1 and 2 subfamily. Genet Resour Crop Evol.

[67] Letunic I, Khedkar S, Bork P (2021). SMART: recent updates, new developments and status in 2020. Nucleic Acids Res.

[68] Saxena L, Iyer BK, Ananthanarayan L (2010). Purification of a bifunctional amylase/protease inhibitor from ragi (*Eleusine coracana*) by chromatography and its use as an affinity ligand. J Chromatogr B Biomed Appl.

[69] Shivaraj B, Pattabiraman TN (1981). Natural plant enzyme inhibitors. Characterization of an unusual α-amylase/trypsin inhibitor from ragi (*Eleusine coracana* Geartn.). Biochem J.

[70] Strobl S, Mühlhahn P, Bernstein R, Wiltscheck R, Maskos K, Wunderlich M, Huber R, Glockshuber R, Holak TA (1995). Determination of the three-dimensional structure of the bifunctional alpha-amylase/trypsin inhibitor from ragi seeds by NMR spectroscopy. Biochemistry..

[71] Boyd DW, Cohen AC, Alverson DR (2002). Digestive enzymes and stylet morphology of *Deraeocoris nebulosus* (Hemiptera: Miridae), a predacious plant bug. Ann Entomol Soc Am.

[72] Terra WR, Ferreira C (1994). Insect digestive enzymes: properties, compartmentalization and function. Comp Biochem Physiol.

[73] Oliva MLV, Silva MCC, Sallai RC, Brito MV, Sampaio MU (2010). A novel subclassification for Kunitz proteinase inhibitors from leguminous seeds. Biochimie..

[74] Sivakumar S, Mohan M, Thayumanavan B (2005). Characterization of insect proteinases and their inhibition by finger and little millet inhibitors. J Plant Biochem Biotechnol.

[75] Sivakumar S, Mohan M, Franco OL, Thayumanavan B (2006). Inhibition of insect pest α-amylases by little and finger millet inhibitors. Pestic Biochem Physiol.

[76] Lüthi C, Alvarez-Alfageme F, Ehlers JD, Higgins TJV, Romeis J (2013). Resistance of αAI-1 transgenic chickpea (*Cicer arietinum*) and cowpea (*Vigna unguiculata*) dry grains to bruchid beetles (Coleoptera: Chrysomelidae). Bull Entomol Res.

[77] Franco OL, Rigden DJ, Melo FR, Bloch C, Silva CP, Grossi de Sá MF (2000). Activity of wheat α-amylase inhibitors towards bruchid α-amylases and structural explanation of observed specificities. Eur J Biochem.

[78] Franco OL, Melo FR, Mendes PA, Paes NS, Yokoyama M, Coutinho MV, Bloch C, Grossi-de-Sa MF (2005). Characterization of two *Acanthoscelides obtectus* alpha-amylases and their inactivation by wheat inhibitors. J Agric Food Chem.

[79] Basi G, Schmid E, Maundrell K (1993). TATA box mutations in the *Schizosaccharomyces pombe* nmt1 promoter affect transcription efficiency but not the transcription start point or thiamine repressibility. Gene..

[80] Bauer D. Variant calling comparison CASAVA1.8 and GATK. Nat Prec. 2011;1:1–7.

[81] Abuín JM, Pichel JC, Pena TF, Amigo J (2016). SparkBWA: speeding up the alignment of high-throughput DNA sequencing data. PLoS One.

[82] Meng L, Li H, Zhang L, Wang J (2015). QTL IciMapping: integrated software for genetic linkage map construction and quantitative trait locus mapping in biparental populations. Crop J.

[83] Bradbury PJ, Zhang Z, Kroon DE, Casstevens TM, Ramdoss Y, Buckler ES (2007). TASSEL: software for association mapping of complex traits in diverse samples. Bioinformatics..

[84] Tamura K, Stecher G, Peterson D, Filipski A, Kumar S (2013). MEGA6: molecular evolutionary genetics version 6.0 analysis. Mol Biol Evol.

[85] Saitou N, Nei M (1987). The neighbor-joining method: a new method for reconstructing phylogenetic trees. Mol Biol Evol.

[86] Bailey TL, Elkan C (1994). Fitting a mixture model by expectation maximization to discover motifs in biopolymers. Proceedings of the Second International Conference on Intelligent Systems for Molecular Biology.

[87] Toni LS, Garcia AM, Jeffrey DA, Jiang X, Stauffer BL, Miyamoto SD, Sucharov CC (2018). Optimization of phenol-chloroform RNA extraction. MethodsX.

[88] Likhite N, Warawdekar UM (2011). A unique method for isolation and solubilization of proteins after extraction of RNA from tumor tissue using trizol. J Biomol Tech.

[89] Chen M, Wu J, Wang L, Mantri N, Zhang X, Zhu Z, Wang S (2017). Mapping and genetic structure analysis of the *anthracnose resistance* locus *co-1*^*HY*^ in the common bean (*Phaseolus vulgaris* L.). PLoS One.

[90] Livak KJ, Schmittgen TD (2001). Analysis of relative gene expression data using real-time quantitative PCR and the 2^-ΔΔCT^ method. Methods..

