# Supplementary material for: QTL mapping and identification of genes associated with the resistance to Acanthoscelides obtectus in cultivated common bean using a high-density genetic linkage map
Source: BMC Plant Biol. 2022 May 25;22:260. doi: 10.1186/s12870-022-03635-4 (PMC9131570; doi:10.1186/s12870-022-03635-4)
Supplement: Supplementary file 1 — Additional file 1. [file 12870_2022_3635_MOESM1_ESM.docx]

**Supplementary table and figure as follow:**


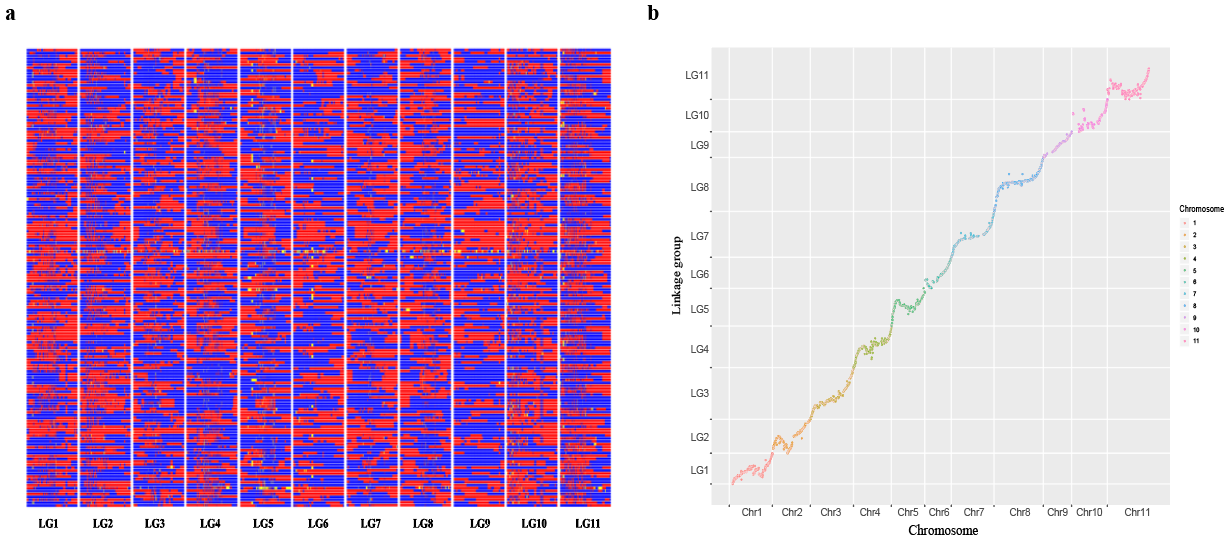


**Supplementary Fig S1** **Recombination map and collinearity analysis of genetic map and physical map.** **(a)** Recombination map of 157 RILs. The vertical axis is different lines in the recombination inbred Line. The horizontal axis is the genetic linkage group. Red means from BKB; Blue comes from LYD3; The yellow is heterozygote. **(b)** Collinearity analysis of the ordinal position of markers on genetic map and physical map. Eleven linkage groups were corresponded to exactly 11 chromosomes, with linkage groups on the vertical axis and chromosomes on the horizontal axis.


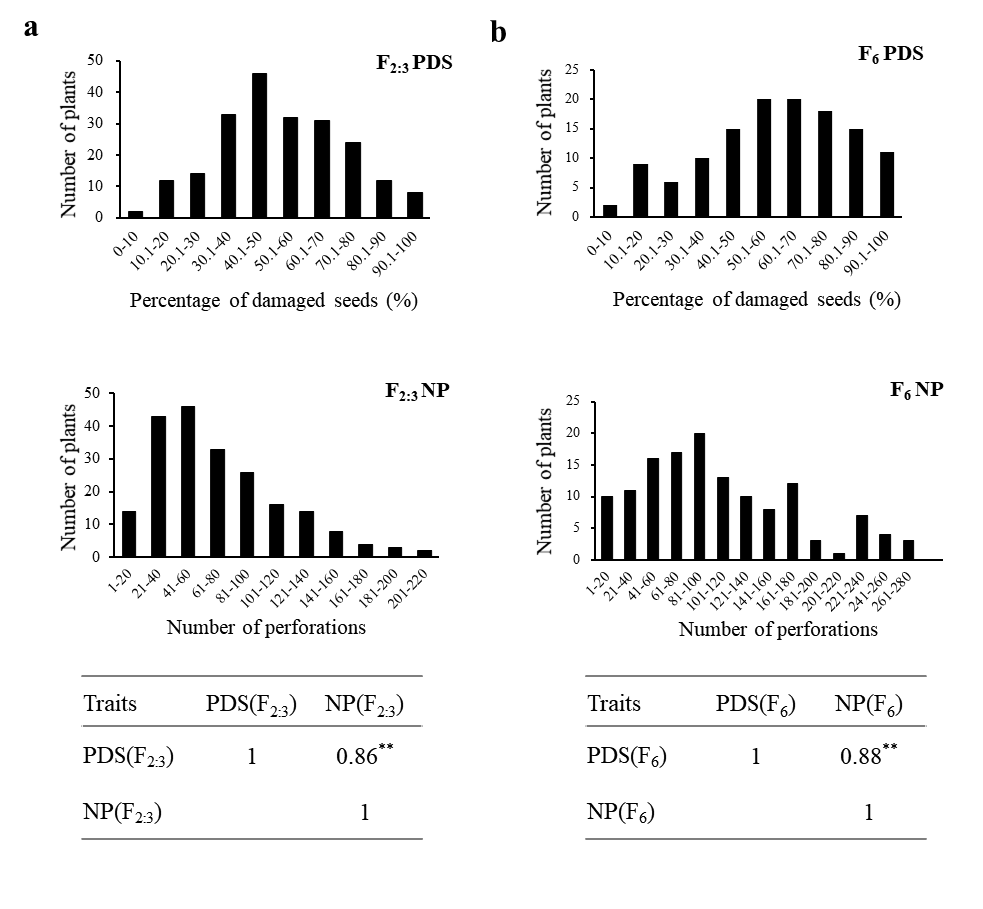


**Supplementary Fig S2** **Frequency distribution of PDS and NP in the F_2:3_ and F_6_ lines. (a)** Frequency distribution of the PDS and NP in F_2:3_ families and the correlation analysis between PDS and NP in F_2:3_ population. **(b)** Frequency distribution of PDS and NP in the F_6_ lines from a cross of LYD3 and BKB, and the correlation analysis between PDS and NP in F_6_ population. PDS represents the percentage of damaged seeds. NP represents the number of perforations. **Significant correlation at *P* < 0.01.


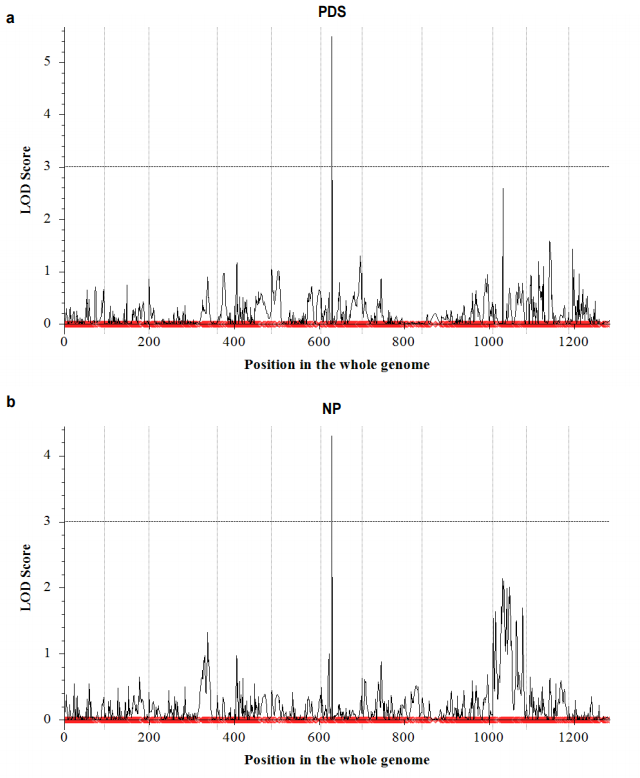


**Supplementary Fig S3** **QTLs associated with resistance to *Acanthoscelides obtectus* (Say) in genome-wide**. The graph showed the LOD of 11 chromosomes suggesting major quantitative trait locus for resistance to *Acanthoscelides obtectus* (Say) in RIL population derived from LYD3 × BKB by inclusive composite interval mapping method. **(a)** QTL for number of PDS (the percentage of damaged seeds). **(b)** QTL for NP (the number of perforations). The horizontal axis represents genetic distances of 11 chromosomes, and the dotted line separates each chromosome. The vertical axis represents the LOD value.


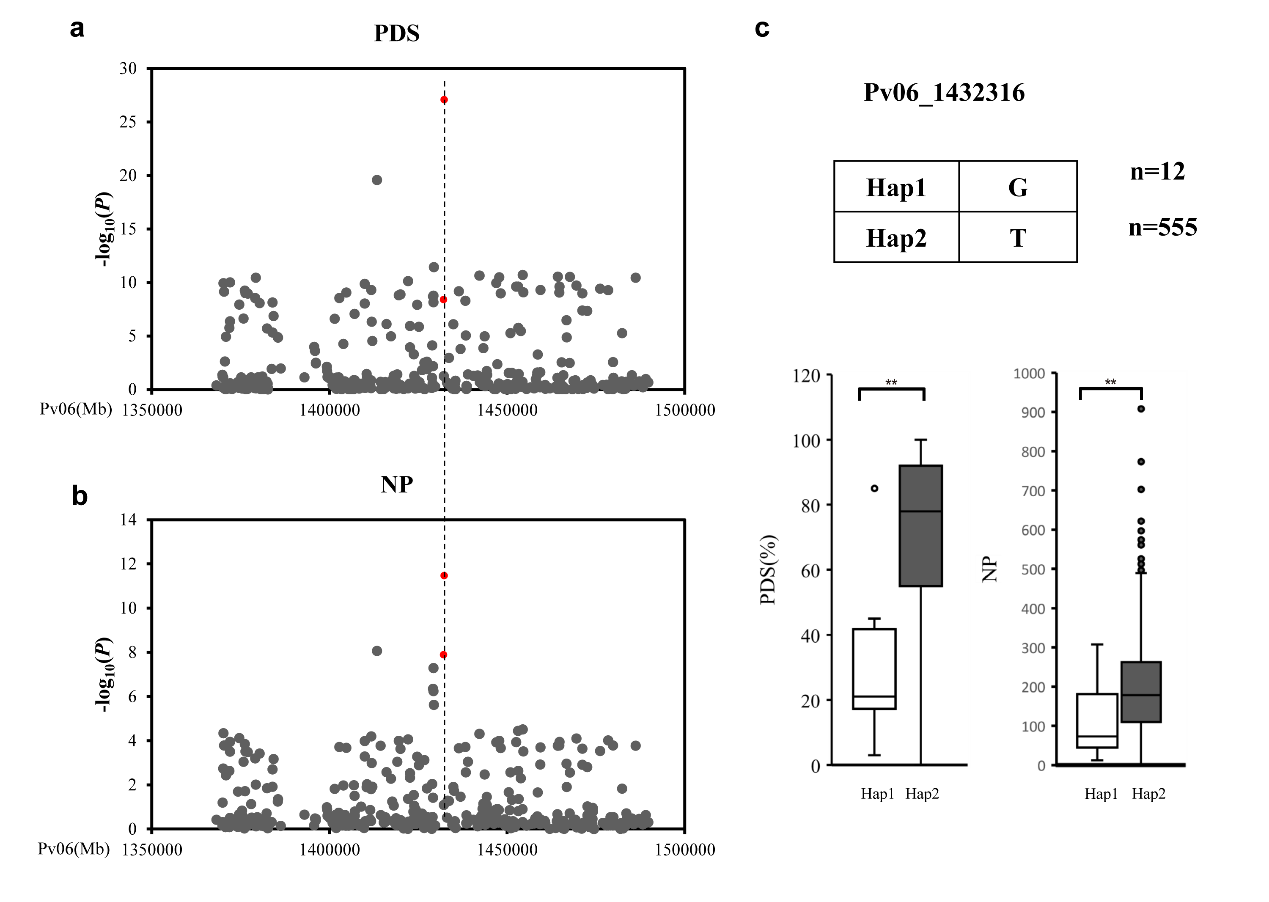


**Supplementary Fig S4** **Manhattan plot of the region of QTL associated with** **the reduced percentage of damaged seeds and the numbers of infected holes on** **chromosome 6**. (a) Local Manhattan plot of the region of QTL associated with the percentage of damaged seeds (PDS). The dotted line indicates the position of a significant SNP (Pv06_1432316). (b) Local Manhattan plot of the region of QTL associated with the numbers of perforations (NP) on chromosome 6. The dotted line indicates the position of a significant SNP (Pv06_1432316). (c) boxplot for percentage of damaged seeds based on the haplotypes (Hap), which were formed by the variation in the marker alleles in Pv06_1432316, and there were significant differences in PDS and NP between the two haplotypes. The middle line indicates the median, the box indicates the range of the 25th and 75th percentiles of total data, the whiskers indicate the interquartile range, and the outer dots are outliers. **indicates p< 0.01, Student’s t-test, two-tailed.

**Supplementary Fig S****5** The coding sequence of gene *Phvul.006G003700* between BKB (resistant) and LYD3 (susceptible). There was no difference between two sequences.


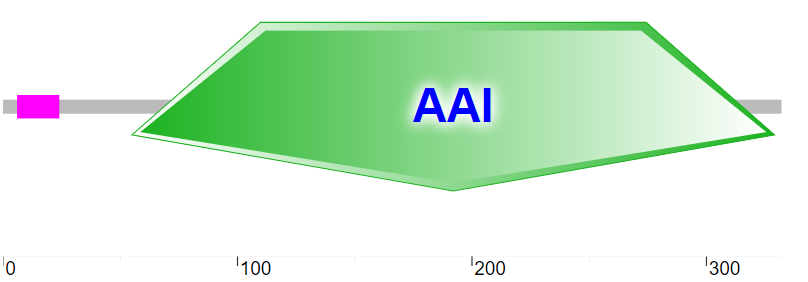


**Supplementary Fig S6** The annotation of Phvul.006G003700 protein domains by searching on SMART database. SMART protein schematic showed a low complexity domain between 6 to 24 amino acid residues (pink box in the figure) and an AAI domain between 55 to 329 amino acid residues (green box in the figure).


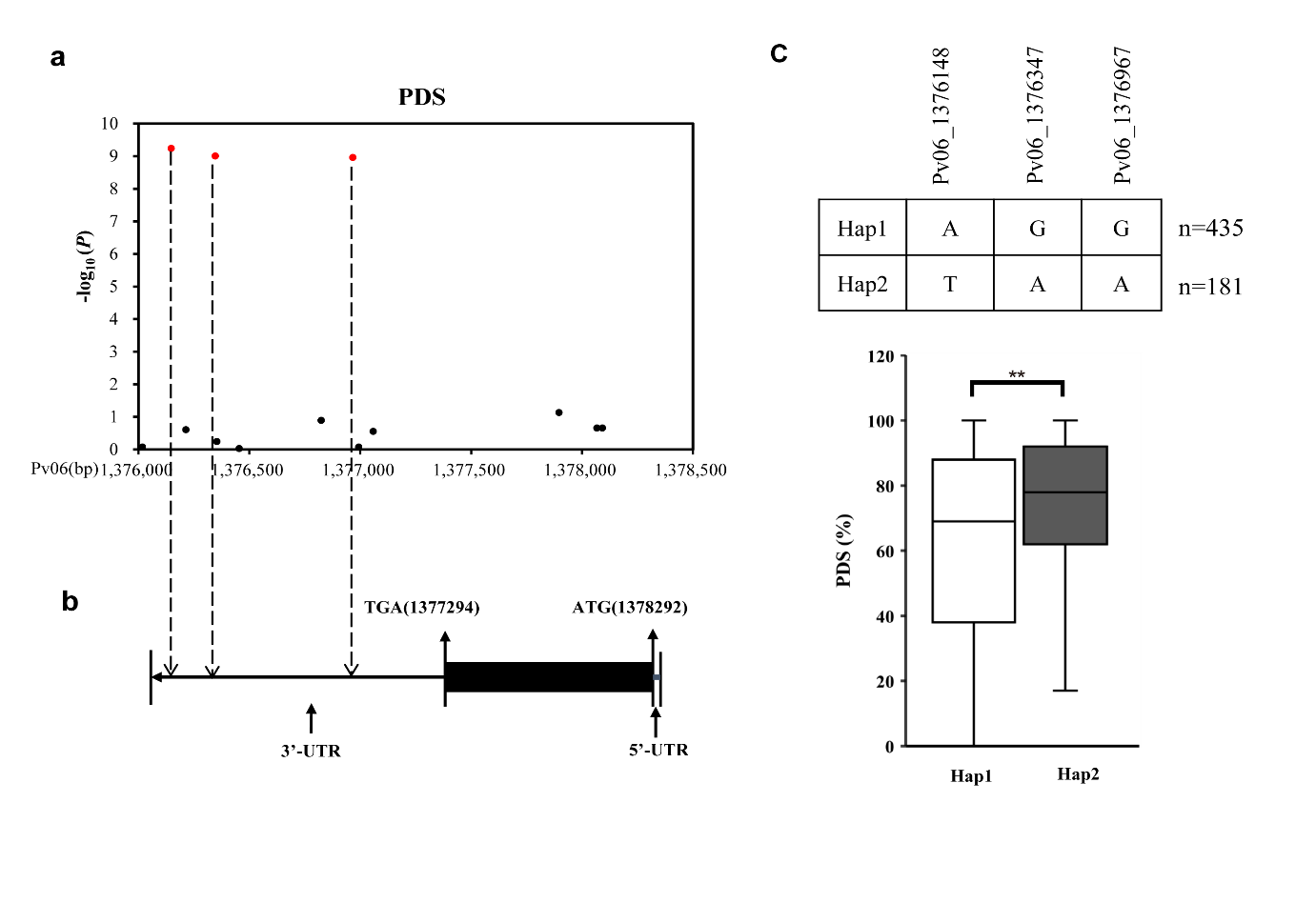


**Supplementary Fig S7** Analysis of significant SNPs associated with the percentage of damaged seeds in candidate gene region. **(a)** Local Manhattan plot of the Phvul.006G003700 region on chromosome 6. The dotted arrow indicates the position of three significant SNPs (Pv06_1376148, Pv06_1376347, Pv06_1376967). **(b)** Gene structure of Phvul.006G003700 (reverse). The filled black box represents the coding sequence. The black lines represent the 5’-UTR and 3’-UTR. **(c)** The boxplot for percentage of damaged seeds based on the haplotypes (Hap), which were formed by the variation in the marker allele at Pv06_1376148, Pv06_1376347 and Pv06_1376967. The middle line indicates the median, the box indicates the range of the 25th and 75th percentiles of total data, the whiskers indicate the interquartile range, and the outer dots are outliers. **indicates p < 0.01, student’s t-test.


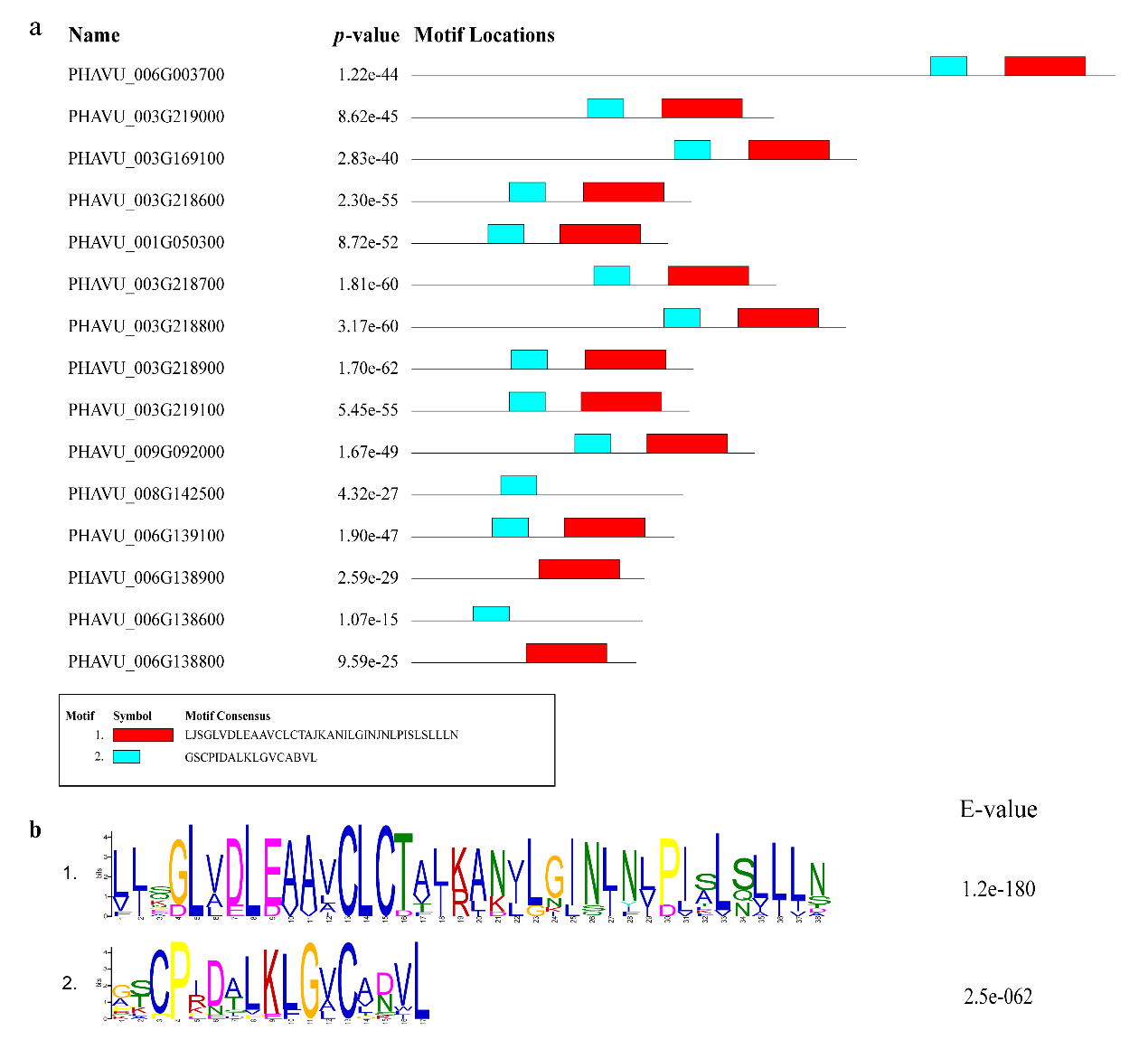


**Supplementary Fig S8** The conservative motifs with the e-value >1E-60 among Phvul.006G003700 and homology proteins in *P*. *vulgaris*. (**a**) The *p*-value and location of conservative motifs in Phvul.006G003700 and homology proteins in *P. vulgaris,* and the consensus sequence of conservative motifs. (**b**) The logo of conservative motifs and the e-value.


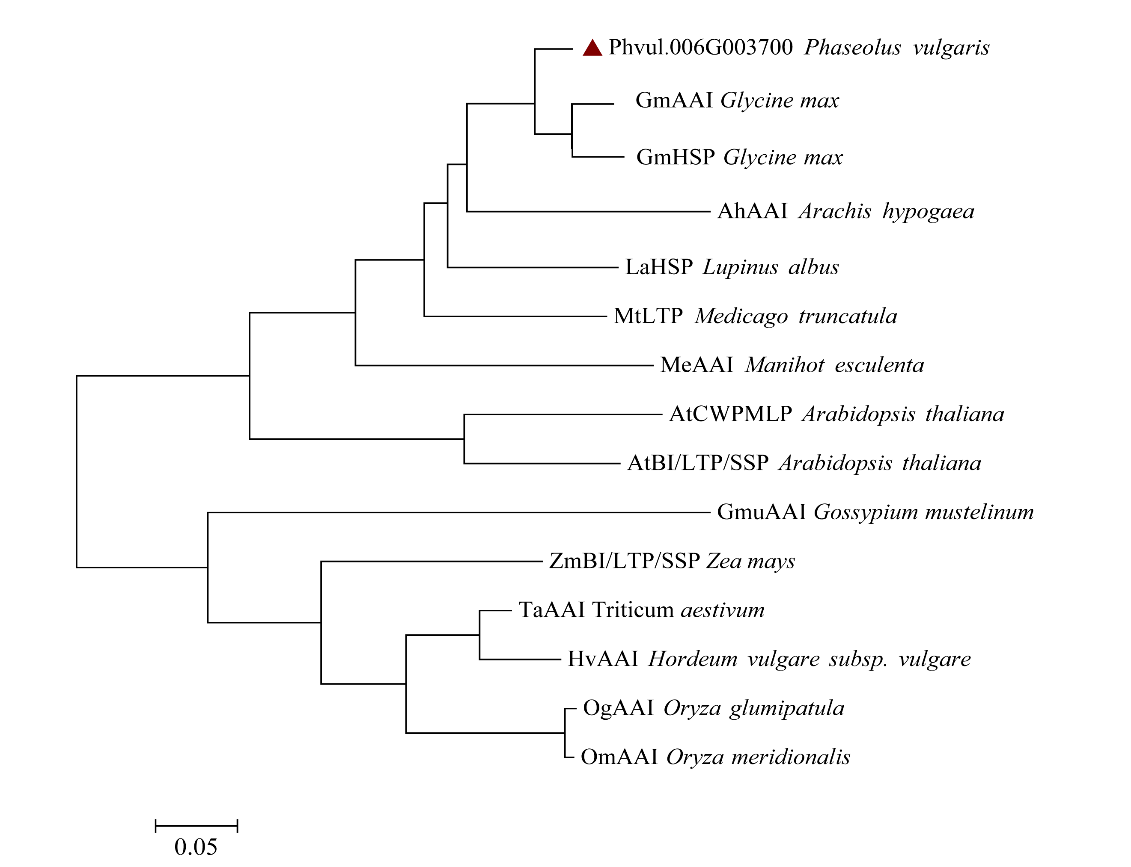


**Supplementary Fig S9** Phylogenetic relationship tree of Phvul.006G003700 with 14 homologous proteins in other crops. The red triangle marker highlights the target gene in common bean.

**
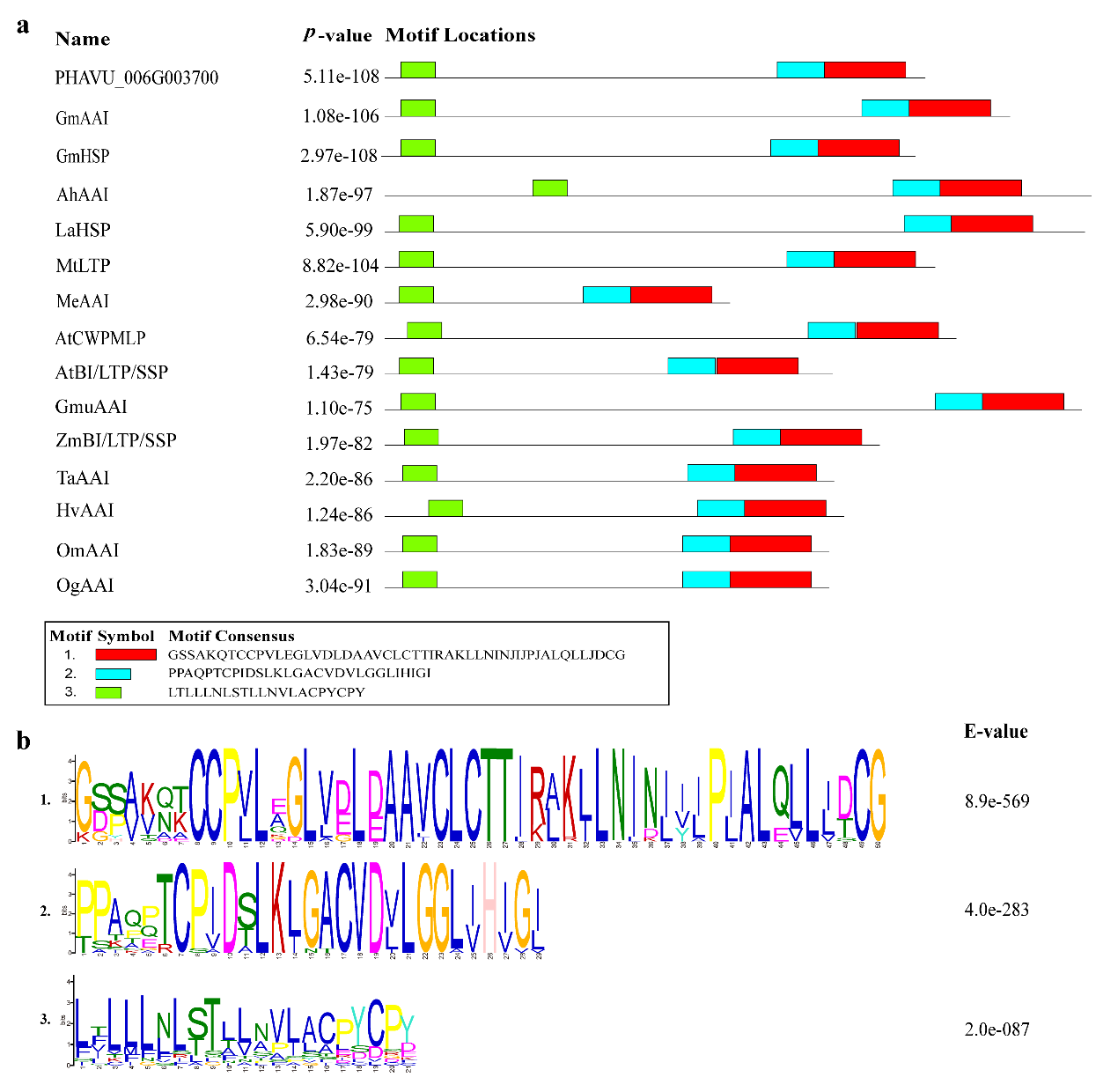
**

**Supplementary Fig S10** The conservative motifs with the e-value >1E-60 among Phvul.006G003700 and 14 homology proteins in other crops. **(a)** The *p*-value and location of conservative motifs in Phvul.006G003700 and homology proteins in other crops, and the consensus sequence of conservative motifs. **(b)** The logo of conservative motifs and the e-value.
